# Live immunisation against *Theileria parva*: containing or spreading the disease?

**DOI:** 10.1016/j.pt.2007.09.002

**Published:** 2007-12

**Authors:** Declan J. McKeever

**Affiliations:** Royal Veterinary College, University of London, Hawkshead Lane, North Mymms, Hertfordshire AL9 7TA, UK

## Abstract

Although a live vaccine against *Theileria parva* has been available for over 30 years, concerns that vaccine strains can become established in resident tick populations have impeded its uptake in endemic areas. Recently, Oura *et al.* have examined the persistence of vaccine strains in immunised cattle using polymorphic genomic markers. They confirm that elements of the vaccine establish a carrier state in vaccinated animals and present evidence that alleles associated with vaccine strains emerge in co-grazing non-vaccinated cattle. However, the epidemiological impact of these observations might be tempered by extensive recombination of co-ingested strains in the tick vector.

## Vaccine-based control strategies for vector-borne diseases

Vector-borne diseases present several challenges for vaccine-based control strategies. These include variations in challenge intensity associated with vector dynamics, emergence of novel pathogen variants in the vector population and the opportunities for immune evasion by the pathogen through its recombination in the vector. Such complexities often complicate the prediction of vaccine performance under field conditions, particularly when the vaccine itself gives rise to transient patent infection. Two recent papers by Oura and colleagues [1,2] provide a powerful insight into these issues with respect to *Theileria parva*, an apicomplexan parasite of cattle transmitted by *Rhipicephalus appendiculatus* ticks. This parasite invades and transforms lymphocytes, causing a severe lymphoproliferative disease known as East Coast fever (ECF) in eastern, central and southern Africa.

## *Theileria parva* and ECF

The life cycle of *T. parva* is similar to that of malaria, with asexual expansion in the mammalian host and a brief sexual phase in the arthropod vector. The major replicating stage is the schizont, which transforms infected lymphocytes and divides in synchrony with them, ensuring transmission of infection to each daughter cell. The pathology associated with ECF is due almost entirely to dissemination of infected lymphoblasts through lymphoid and non-lymphoid tissues. Merozoites released from a proportion of infected cells after further differentiation of the schizont give rise to transmissible intraerythrocytic piroplasms. Ingestion of piroplasms by a subsequently feeding tick results in the formation of gametes and sexual recombination, which give rise ultimately to cattle-infective sporozoite forms in the tick salivary gland.

Cattle can be immunised against *T. parva* by simultaneous inoculation of live sporozoites and long-acting formulations of oxytetracycline – widely known as ‘infection and treatment’ [3,4]. There is strong evidence that protection is mediated by parasite-specific major histocompatibility complex (MHC) class I-restricted cytotoxic T lymphocytes (CTLs), which target the schizont-infected lymphoblast [5,6]. Because it does not take effect until the schizont parasitosis is established, the CTL response does not prevent infection [6]. In addition, recovered cattle – and those immunised by infection and treatment – are almost invariably long-term carriers of piroplasm forms [7,8]. Because piroplasms of *T. parva* undergo only limited replication [9] the carrier state probably arises from persistence of small numbers of schizont-infected lymphoblasts.

## Parasite diversity and existing vaccines

A key feature of the epidemiology of *T. parva* and ECF is the diversity of parasite populations in the field. This is evident at both antigenic [10,11] and molecular [12–14] levels and results commonly in a lack of cross-protection between distinct isolates of the parasite [10,15]. Nonetheless, broad protection can be obtained with immunisation by infection and treatment using relatively few parasite stocks, suggesting that antigenic variation might be limited [4]. Although several *T. parva* isolates have been evaluated for infection and treatment immunisation, the formulation used most widely is the original trivalent vaccine that was developed by Radley *et al.* in 1975 [4]. Known as the Muguga Cocktail, the formulation comprises three stocks of the parasite – Muguga, Kiambu 5 and Serengeti-transformed – and protects against a range of geographically disparate isolates. The vaccine has been deployed extensively in Tanzania [16] and Uganda [17] and, to a lesser extent, in Malawi and southern Zambia.

Diversity of *T. parva* parasites in the field is also reflected in the virulence phenotype: some isolates are highly pathogenic, whereas others give rise to only mild infections [18]. In addition, the duration of the carrier state varies following infection with different isolates [19]. This phenotypic variation and associated concerns over the consequences of the large-scale introduction of parasite strains into areas free of them previously have been significant obstacles to widespread uptake of immunisation by infection and treatment in some ECF-endemic countries. Fundamental to these concerns is the question of whether vaccine strains become established in the resident tick populations and transmit to unvaccinated animals.

## Molecular tracking of parasite populations following vaccination

Oura *et al.* have now provided a powerful insight into this question by tracking individual components of the Muguga Cocktail in cattle immunised by infection and treatment under field conditions in Uganda [1,2]. This was achieved by PCR amplification of genomic DNA from blood using a panel of parasite micro- and mini-satellite markers (Box 1). Based on the numbers of alleles observed with two of the markers in the composite stabilate, at least six genotypes are present in the Muguga Cocktail [2]. Moreover, analysis of samples taken from 14 calves 2 years previously, on day 17 after immunisation, revealed patent infections in all but two of the animals using both markers, with each calf harbouring one to three distinct genotypes [2]. All six alleles of each marker were represented among the calves, along with an additional product observed in one animal that was not present in the stabilate. This analysis confirms that the Muguga Cocktail is complex genotypically and capable of establishing multiple acute infections in individual animals.

The group went on to evaluate the persistence of parasites derived from each component of the cocktail in immunised animals. Alleles of the two discriminatory microsatellite markers were allocated to each component by comparing marker profiles of the stabilate with those of single cell lines derived from each of the constituent stocks [1]. However, because each line contained only one genotype, it was possible to account for only three out of the six alleles at either locus. Hence, only one allele at each locus could be assigned conclusively to individual component stocks, each of which could theoretically account for up to four out of the six genotypes. Nonetheless, it was possible to track the persistence of three of the cocktail genotypes in immunised cattle, with one representing each of the component stocks. No evidence was found for the persistence of the genotypes representing the Muguga or Serengeti-transformed components in animals vaccinated previously and, in 13 calves undergoing immunisation, these were undetectable in all but one animal after 48 days [1]. By contrast, the Kiambu 5-derived genotype persisted in 70% of cattle, often along with other presumably local genotypes, in some cases 4 years after vaccination [2]. The transient persistence of the Muguga-derived element is in line with previous observations of this stock [19,20]. Given that the genotype associated with the Serengeti-transformed component shares 27 out of 31 marker alleles with Muguga [1], its predominantly brief carrier state is perhaps also unsurprising. The close relatedness of these two genotypes has been reported previously [21] but is inconsistent with the initial cross-protection studies [3,4] that led Radley *et al.* to include it in the cocktail [4]. As suggested by Oura *et al.* [1], it is possible that the original Serengeti-transformed stock (or elements of it) has been lost in the course of passage. Alternatively, the stock might contain additional genotypes (perhaps represented in the unassigned alleles present in the cocktail), which contribute to the breadth of protection engendered by the composite vaccine.

## Transmission of vaccine components to unvaccinated animals

The formal demonstration by Oura *et al.* in these studies that at least some of the genotypes present in the Muguga Cocktail establish a persistent carrier state in immunised cattle confirms that vaccine strains become accessible to the local tick population and might therefore transmit to co-grazing animals. This possibility was examined by analysing blood taken from unvaccinated cattle on the same farm with several markers that distinguish the Kiambu 5-derived genotype detected in carrier vaccinates [2]. For each marker, the associated allele was detected in four out of 13 animals sampled, along with additional alleles, derived presumably from local stocks. This was interpreted as strong evidence that the genotype had been transmitted to these animals from carrier-vaccinated cattle. Although transmission almost certainly occurred, the notion that *Theileria* strains are maintained intact in cattle populations has been challenged recently [22]. Using a panel of 64 markers, including some of those described by Oura *et al.* [1,2], a stabilate of the Marikebuni isolate of *T. parva* was genotyped before and after calf and tick passage. By genotyping clones derived from parent and daughter populations, evidence was found for substantial recombination during the sexual stages of the life cycle. The parent stabilate was complex genotypically, with 48 genotypes observed among 231 clones analysed and one accounting for 75% of the population. Recombination was evident from the apparent reassortment of alleles in blocks and substantial inbreeding in relation to the dominant clone [22]. Furthermore, several new variants of each parasite chromosome were observed in the daughter stabilate and, although the dominant genotype still accounted for a high proportion of the clones, none of the other components of the parent population were present. Inbreeding with respect to the dominant genotype was considerably greater [22]. These observations suggest that *T. parva* strains within complex populations are ephemeral, with alleles reassorting in different combinations during each tick passage. Given the multiple carrier states observed by Oura *et al.* [1,2], it seems unlikely that strains present in the Muguga Cocktail would escape into the resident tick population as intact genotypes and persist in the area.

Nonetheless, the results of Oura *et al.* [1,2] do confirm that alleles derived from the Muguga Cocktail can transmit to unvaccinated cattle and become incorporated into the resident parasite gene pool. The epidemiological significance of this remains unclear. Based on a distinct molecular analysis, Geysen *et al.* [23] suggested that the Muguga and Serengeti-transformed components of the vaccine were prevalent widely among field cases of ECF in the Southern Province of Zambia, following deployment of the Muguga Cocktail over a 7 year period. This now seems unlikely, given the observed absence of these components from the carrier animals studied by Oura *et al*. [1,2]. In any event, establishment of Kiambu 5 alleles in the resident parasite population of the farm studied appears to have been uneventful in terms of disease [2]. The animals remained under persistent challenge, despite regular acaricide treatment, as evidenced by the presence of multiple parasite genotypes in unvaccinated animals and a history of severe ECF before implementation of vaccination [1,2].

## Concluding remarks and future directions

Perhaps a more important question relates to the genotypic composition of the stocks that make up the Muguga Cocktail and the possibility that elements could be lost over time. The former is fundamental to the efficacy of the vaccine and is likely to change with recombination during passage of each stock. Consequently, a rigorous definition of the genotypes present in the Muguga, Kiambu 5 and Serengeti-transformed stocks that comprise the current Muguga Cocktail vaccine stabilate appears timely. This would enable the precise identification of those components responsible for the breadth of coverage conferred by the vaccine and, through the establishment of cloned stocks, ensure their continued presence in future batches.

## Box 1 Genotyping with micro- and mini-satellites

Micro- and mini-satellites are stretches of tandemly repeated nucleotide sequences that are dispersed throughout eukaryotic genomes, both within genes and in non-coding regions. Microsatellites generally comprise short tandem repeats (∼2–6 bp), whereas mini-satellites are made up of longer repeating units (∼8–100 bp). Satellite loci evolve rapidly through a combination of replication slippage and meiotic recombination and are often selectively neutral. As a result, the number of repeated units occurring at individual satellite loci varies greatly within populations and constitutes a powerful means of genetic fingerprinting. This is achieved by PCR amplification of the repeated region using primers based on flanking sequences. The precision of the fingerprint obtained is a function of the number of satellite loci examined across the genome in question.
